# Habitual fish intake and clinically silent carotid atherosclerosis

**DOI:** 10.1186/1475-2891-13-2

**Published:** 2014-01-09

**Authors:** Silvio Buscemi, Antonio Nicolucci, Giuseppe Lucisano, Fabio Galvano, Giuseppe Grosso, Serena Belmonte, Delia Sprini, Silvia Migliaccio, Luisella Cianferotti, Maria Luisa Brandi, Giovam Battista Rini

**Affiliations:** 1 Dipartimento Biomedico di Medicina Interna e Specialistica (DIBIMIS) – Laboratorio di Nutrizione Clinica, University of Palermo, Palermo, Italy; 2 Dipartimento di Farmacologia Clinica ed Epidemiologia, Consorzio Mario Negri Sud, S. Maria Imbaro, Mozzagrogna Chieti, Italy; 3 Dipartimento di Scienze del Farmaco, University of Catania, Catania, Italy; 4 Dipartimento di Medicina Sperimentale – “Sapienza” University Roma, Roma, Italy; 5 Dipartimento di Medicina Interna, University of Firenze, Firenze, Italy

**Keywords:** Fish consumption, Carotid atherosclerosis, Carotid intima-media thickness, Hypertension, Pulse pressure

## Abstract

**Background:**

Fish consumption is recommended as part of a healthy diet. However, there is a paucity of data concerning the relation between fish consumption and carotid atherosclerosis. We investigated the association between habitual fish consumption and asymptomatic carotid atherosclerosis, defined as the presence of plaques and/or increased intima-media thickness (≥ 0.90 mm), in non-diabetic participants.

**Methods:**

Nine hundred-sixty-one (range of age: 18–89 yrs; 37.1% males) adult participants without clinically known atherosclerotic disease were randomly recruited among the customers of a shopping mall in Palermo, Italy, and cross-sectionally investigated. Each participant answered a food frequency questionnaire and underwent high-resolution ultrasonographic evaluation of both carotid arteries. Routine laboratory blood measurements were obtained in a subsample of 507 participants.

**Results:**

Based on habitual fish consumption, participants were divided into three groups: non-consumers or consumers of less than 1 serving a week (24.0%), consumers of 1 serving a week (38.8%), and consumers of **≥** 2 servings a week (37.2%). Age-adjusted prevalence of carotid atherosclerosis (presence of plaques or intima media thickness ≥ 0.9 mm) was higher in the low fish consumption group (13.3%, 12.1% and 6.6%, respectively; P = 0.003). Multivariate analysis evidenced that carotid atherosclerosis was significantly associated with age (OR = 1.12; 95% CI = 1.09-1.14), hypertension on pharmacologic treatment (OR = 1.81; 95% CI = 1.16-2.82), and pulse pressure (OR = 1.03; 95% CI = 1.01-1.04), while consuming **≥**2 servings of fish weekly was protective compared with the condition of consumption of <1 serving of fish weekly (OR = 0.46; 95% CI = 0.26-0.80).

**Conclusions:**

High habitual fish consumption seems to be associated with less carotid atherosclerosis, though adequate interventional trials are necessary to confirm the role of fish consumption in prevention of cardiovascular disease.

## Background

Cardiovascular disease (CVD) is the leading cause of morbidity and mortality in the Western world [1]. Fish consumption is advised as part of a healthy diet to reduce the risk of cardiovascular diseases [2-4]. Different mechanisms by which fish consumption influences the pathways leading to atherosclerosis have been identified, and n-3 polyunsaturated fatty acids (n-3 PUFA) have been indicated as the substance contained in fish that is most active in cardiovascular protection. Nonetheless, there are still some controversial issues to be settled [5,6]. Consumption of fish has been found to be protective against coronary heart disease [7], but data for protection against stroke are less convincing [8,9]. There is a substantial body of literature demonstrating a favourable association of fish intake and/or n-3 PUFA with subclinical atherosclerosis. However, some uncertainty still remains, and some studies that have evaluated the effects of fish or n-3 PUFA intake on subclinical atherosclerosis in animal models [10-12], and in humans [13-15], have been inconsistent in results and conclusions.

Regardless of whether it is asymptomatic, carotid atherosclerosis has important clinical implications. In fact, ultrasound-assessed plaques are independent predictors of cardiovascular events [16], and carotid intima-media thickness (c-IMT) is a well-validated surrogate marker of future coronary events [17].

We hypothesized that fish consumption would be inversely associated with asymptomatic carotid atherosclerosis (plaques and/or increased c-IMT). Therefore, we investigated the association between fish consumption and asymptomatic carotid atherosclerosis in a group of non-diabetic, randomly selected, adults with no known atherosclerotic cardiovascular disease.

## Methods

This observational, cross-sectional study was carried out in Palermo, the largest city in Sicily, Italy, with a population of 663,173. From March 28^th^ to April 10^th^, 2011, groups composed of physicians (n = 5) and dieticians (n = 13) alternated their presence inside the *Forum*, a shopping mall in Palermo, from 9:00 a.m. until 9:00 p.m. and investigated those customers who asked to participate in the investigations proposed in an announcement presented on posters placed inside the *Forum*.

The *Forum* is the largest shopping center in Palermo, and customers come from all parts of the city, suburbs and neighboring areas. Data provided by the *Forum* administration show that the characteristics of their habitual customers were heterogeneous in terms of gender (females 65%, males 35%), age (10–54 years 50%, > 55 years 50%), place of residence (Palermo 62%, outside of Palermo 38%), education (college graduates = 14%, high school graduates = 37%, lower secondary school = 32%, primary school = 17%), and employment status (housewife = 40%, retired = 23%, employed = 19%, student = 8%, unemployed = 6%, manager/professional = 4%,).

Inclusion criteria were participants aged ≥ 18, and with residence in Palermo. There was no incentive provided to the participants. In order to promote the participation of people of younger age without known cardiovascular, metabolic or nutritional diseases an echographic check of the thyroid was also proposed to the customers of the mall. Further details about patient recruitment procedures have been presented elsewhere [18].

Participants were asked to come to the Laboratory of Clinical Nutrition of the Department of Internal and Specialized Medicine of the University of Palermo in the following weeks, up until July 15, 2011, to undergo blood sampling for assessment of blood chemistry and hormonal parameters. A blood sample was frozen and stored at -80°C, and a sample was treated and stored for subsequent measurements.

The institutional ethics committee approved the study protocol. Each participant signed an approved informed consent form.

Participants were administered a questionnaire on demographic characteristics, the presence of chronic diseases and pharmacologic treatment, physical activity, including items concerning the level of physical activity and its weekly frequency, daily time watching television, on the computer, and playing video games. Food intake was assessed and included questions on the usual frequency of fish consumption. Concerning habitual fish consumption, the following specific questions were used:

Question: *Do you habitually eat fish or shell-fish (referred to the last 12 months)?*

Answer (sign the one of the following): *never*, *seldom (less than once a week)*, *yes*

Question*: If yes, how many times a week do you estimate you eat fish or shell-fish?*

Answer: ______ times a week.

Question: *If you habitually eat fish, what percentage (from 1% to 100%) of each of the following modalities of stored fish do you eat (please observe that the sum of all items must add up to 100)?*

Answer: A) *Frozen fish ____%* B) *Local fresh fish ____%* C) *Canned fish ____% (A + B + C = 100%).*

Habitual fish intake was categorized as follows: no habitual consumption or less than 1 serving a week = 0, 1 serving a week = 1, more than 1 serving a week = 2. Data requested referred to the last year.

### Measurements

Height and body weight were measured with participants lightly dressed and without shoes (SECA); the body mass index (BMI) was calculated as body weight (kg)/height^2^ (m^2^). Body circumferences were measured at the umbilicus (waist circumference) and at the most prominent buttock level (hip circumference); the ratio (waist-to-hip ratio) was used as an indirect index of body fat distribution.

Systolic and diastolic arterial blood pressure (two measurements obtained at 5-minute intervals in seated position) and heart rate were measured by physicians or dietitians according to standardized procedures (Omron M6; Omron Healthcare Co; Matsusaka, Mie, Japan) after 15 minutes of rest in sitting position; pulse pressure was calculated as the difference between systolic and diastolic blood pressure.

### Carotid intima-media thickness

Images of the right and left extracranial carotid artery walls were obtained in several projections by using a high-resolution ultrasonographic 10-MHz linear array probe (Sonoline G50; Siemens, Germany). The end-diastolic c-IMT of the far wall of both common carotid arteries was measured 10 mm caudal to the bulb, using two-dimensional longitudinal sections of the vessel and the distance from the first echogenic line to the second echogenic line (three values for each carotid artery using antero-posterior, laterolateral and postero-anterior scans); the highest value was considered for calculations [19]. Two physicians were responsible for carrying out the carotid ultrasonographic examination, and were blinded to participants’ characteristics. The intra-observer coefficient of variations were, respectively, 1.2 and 1.1%; the inter-observer coefficient of variation was 2.9%. Previously unknown asymptomatic carotid atherosclerosis was diagnosed in the presence of c-IMT ≥ 0.9 mm and/or plaques in common carotid, carotid bifurcation, extra-cranial internal and external carotid arteries. Carotid plaque was defined as a focal thickness of >1.2 mm [20].

### Laboratory analysis

Capillary blood glucose concentrations were randomly assessed using a glucose reflectometer (Glucocard G meter; Menarini Diagnostics; Florence, Italy). Fasting plasma glucose (FPG), total cholesterol, high-density lipoproteins (HDL) cholesterol, triglicerides, uric acid and creatinine concentrations were ascertained with standard clinical chemistry methods (Glucosio HK UV; Colesterolo tot. Mod P/D; Colesterolo HDL gen 3 mod P/917; Trigliceridi; Acido urico MOD P/917; Creatinina enzimatica; Roche diagnostics, Monza, Italy). Basal insulin concentrations (Elecsys insulina; Roche diagnostics; Monza, Italy) and glycated hemoglobin (HbA_1_c; HbA_1_c gen.3; Roche diagnostics; Monza, Italy) were also measured. Low-density lipoprotein (LDL) cholesterol concentration was calculated by means of Friedewald’s formula [21].

Glomerular filtration rate (GFR) was calculated according to modification of diet in renal disease study (MDRD) [22] and Cockcroft-Gault [23] equations. The HOMA-IR was calculated as described by Matthews et al. [24]. The quantitative insulin sensitivity check index (QUICKI) was calculated as described by Katz et al. [25].

### Statistical analysis

Participant characteristics were grouped in three classes according to fish intake (no habitual consumption or less than 1 serving a week = 0; 1 serving a week = 1; and more than 1 serving a week = 2). Since the three groups were heterogeneous in terms of age, comparisons were done with a generalized linear ANCOVA model for binary, multinomial, and continuous variables that were adjusted for age. Data were therefore reported as estimated means ± SEE for continuous variables, and estimated percentages for categorical ones. Dietary patterns were defined with an *a posteriori* approach by means of cluster analysis, as described elsewhere (18). Briefly, this procedure is based on the intercorrelations among food groups or nutrients, and is not biased because it does not require as a starting point any technical decision on which foods or nutrients are or are not healthy.

Multivariate logistic regression analyses were done to evaluate factors associated with asymptomatic carotid atherosclerosis (plaques and/or increased c-IMT). The following baseline covariates were tested: age (y), gender (male, female), smoking status (former, current or never a smoker), frequency of fish intake (<1, 1, or ≥2 servings/week), physical activity level (light, moderate/heavy or none), use of statins (yes or no), hypertension on treatment (yes or no), and pulse pressure (mmHg). Results of the logistic models are expressed as adjusted odds ratios (ORs) with their 95% confidence intervals. A two-tailed *P* value of < 0.05 was considered significant. All statistical analyses were done using SAS version 9.2 (SAS Institute Inc; Cary, NC, US).

## Results

A total of 1,231 (465 males and 766 females) participants were evaluated; 270 participants were excluded due to the presence of diabetes (type 1 or 2), clinically known atherosclerotic diseases (coronary heart disease, previous stroke, carotid or peripheral atherosclerosis), chronic renal failure, or incomplete anthropometric or carotid measurements. Laboratory blood measurements were obtained in 507 participants.

Men had a higher prevalence of both increased c-IMT (26.8 vs 15.4%, P < 0.001) and presence of carotid plaque (19.8 vs 12.3%; P = 0.002) than women. Based on habitual fish consumption, the remaining 961 (37.1% male) participants were divided into three groups: non-consumers or consumers of less than 1 serving a week (n = 231, 24.0%), consumers of 1 serving a week (n = 373, 38.8%), and consumers of 2 or more servings a week (n = 357, 37.2%). Demographic, anthropometric and clinical characteristics of the three groups of fish consumers are reported in Tables 1 and 2. Based on interviews with participants included in the study, the storage characteristics of habitually consumed fish were in 69% of cases fresh fish of the Mediterranean Sea, in 21% frozen fish, and in 10% canned fish of unspecified origin. Age (range of the sample: 18–89) was higher in the group who habitually consumed more servings of fish (47 ± 14, 48 ± 14, 50 ± 15 years; P < 0.01). The prevalence of clinically silent carotid atherosclerosis was significantly different (P = 0.003) among the three groups, being higher in the low (<1 servings/week) fish consumption group; however, c-IMT was not significantly different among the three groups (P = 0.22) (Table 2). A diet that could be defined as Unhealthy was identified in 21.3% of the cohort (n = 204), and was characterized by high consumption of soft drinks, fried foods, seed oils, cured meats, butter, red meat and sweets. Thirty-four percent of the cohort (n = 329) exhibited a dietary pattern that resembled the Mediterranean Diet, characterized by high intakes of fruit, milk and cheese, olive oil, vegetables, pasta and bread. An intermediate dietary pattern was found in the remaining 44.5% (n = 428) of the cohort. Habitual fish consumption was not significantly different between the 3 dietary patterns (P = 0.09). Multivariate analysis (Table 3) showed that previously unknown clinically silent carotid atherosclerosis was associated with age, hypertension on pharmacologic treatment, and pulse pressure, while consuming 2 or more servings of fish weekly was protective when compared with the condition of consumer of <1 serving of fish weekly. Biochemical blood concentrations, basal insulinemia and HOMA-I and QUICKI were comparable in the three groups (Table 2).

**Table 1:** **Demographic and clinical characteristics of the cohort categorized according to the frequency of consumption of fish**^**1**^

	**Frequency of fish intake (number of servings per week)**			
	**< 1**	**1**	**≥ 2**	**P**^**2**^
	**(n = 231)**	**(n = 373)**	**(n = 357)**	
Gender (% M)	31.2	41.3	36.8	0.06
Education (%)				0.11
0-5 y	7.8	7.0	8.2	
6-8 y	37.6	39.0	34.0	
9-13 y	45.3	41.1	39.7	
> 13 y	9.2	12.9	18.0	
Marital status (%)				0.01
Single	12.4	6.3	11.3	
Married	78.6	89.3	84.5	
Divorced	6.9	3.1	2.7	
Widow/er	2.2	1.3	1.5	
Offspring (%)				0.01
0	14.5	11.8	20.2	
1	15.2	14.3	21.5	
2	51.3	53.6	40.9	
3	19.0	20.4	17.4	
Smoking (%)				0.11
Never smoked	52.8	58.7	60.3	
Former smoker	26.3	21.0	17.0	
Current smoker	20.9	20.2	22.8	
Employment (%)				0.24
Unemployed	58.4	56.8	64.8	
Employed	34.5	36.8	28.8	
Manager/Professional	7.2	6.4	6.4	
Participants on anti-hypertensives (%)	14.4	23.0	13.6	0.002
Use of anti-hypertensives (%)				
Diuretics	5.7	6.6	5.0	0.58
Beta-blockers	7.9	6.9	5.7	0.52
Other anti-hypertensives	7.4	17.6	9.9	<0.001
Use of statins (%)	6.2	3.7	3.6	0.19
Physical activity				0.29
None	52.0	49.6	47.7	
Light	34.3	33.9	40.0	
Moderate/Heavy	13.7	16.5	12.3	

^1^All data are reported as age-adjusted percentages.

^2^Generalized ANCOVA.

**Table 2:** **Anthropometric, echographic and laboratory data of the cohort categorized according to frequency of consumption of fish**^**1**^

	**Frequency of fish intake (number of servings/week)**			
	**< 1**	**1**	**≥ 2**	**P**^**2**^
	**(n = 231)**	**(n = 373)**	**(n = 357)**	
Age (years)	47 ± 14	48 ± 14	50 ± 15	0.003
Body weight (kg)	72.1 ± 1.1	74.6 ± 0.8	73.6 ± 0.9	0.17
BMI (kg/m^2^)	27.6 ± 0.3	28.0 ± 0.3	27.9 ± 0.3	0.58
Circumferences:				
Waist (cm)	93.6 ± 0.9	94.8 ± 0.7	94.2 ± 0.7	0.57
Hip (cm)	104.8 ± 0.8	104.2 ± 0.6	105.0 ± 0.7	0.66
Waist-to-hip ratio	0.89 ± 0.06	1.03 ± 0.05	0.92 ± 0.05	0.14
Systolic BP (mmHg)	129 ± 1	129 ± 1	130 ± 1	0.52
Diastolic BP (mmHg)	79 ± 1	78 ± 1	78 ± 1	0.67
Pulse pressure (mmHg)	50 ± 1	51 ± 1	52 ± 1	0.20
Heart rate (beats/min)	75 ± 1	74 ± 1	74 ± 1	0.18
c-IMT (mm)	0.63 ± 0.01	0.66 ± 0.01	0.65 ± 0.01	0.22
c-IMT ≥0.9 mm, (%)	13.2	12.0	6.5	0.002
Carotid plaques, (%)	10.1	9.2	6.1	0.06
Carotid atherosclerosis^3^, (%)	13.3	12.1	6.6	0.003
Random capillary blood glucose (mg/dl)	89 ± 1	89 ± 1	87 ± 1	0.95
	n = 221	n = 353	n = 342	
Blood concentration of	n = 124	n = 216	n = 167	
Glycated hemoglobin (%)	5.6 ± 0.04	5.6 ± 0.03	5.6 ± 0.03	0.63
Glucose (mg/dL)	90 ± 1	91 ± 1	90 ± 1	0.95
Total cholesterol (mg/dL)	212 ± 3	210 ± 3	217 ± 3	0.25
HDL cholesterol (mg/dL)	59 ± 1	59 ± 1	61 ± 1	0.21
Triglycerides (mg/dL)	103 ± 4	101 ± 3	100 ± 4	0.86
LDL cholesterol (mg/dL)	133 ± 3	132 ± 2	136 ± 3	0.51
Uric acid (mg/dL)	4.8 ± 0.1	5.1 ± 0.1	4.9 ± 0.1	0.08
Insulin (μU/mL)	10.0 ± 0.5	9.3 ± 0.4	9.5 ± 0.5	0.65
HOMA-I	2.29 ± 0.14	2.14 ± 0.11	2.18 ± 0.12	0.69
QUIKI	0.35 ± 0	0.35 ± 0	0.35 ± 0	0.73
Creatinine (mg/dL)	0.82 ± 0.02	0.85 ± 0.01	0.82 ± 0.02	0.33
GFR				
Cockorft-Gault (mL/min)	104.8 ± 2.5	105.4 ± 2.0	105.8 ± 2.2	0.95
MDRD (mL/min/1.73 m^2^)	93.2 ± 1.6	91.2 ± 1.2	93.2 ± 1.4	0.47

^1^All data are reported as age-adjusted estimates means ± SE or percentages.

^2^Generalized ANCOVA.

^3^c-IMT ≥0.9 mm and/or presence of carotid plaque.

BMI, body mass index; BP, blood pressure; GFR, glomerular filtration rate; HDL, high-density lipoproteins; HOMA-I, homeostasis model assessment of insulin resistance; c-IMT, carotid intima-media thickness; MDRD, Modification of Diet in Renal Disease Study; LDL, low-density lipoproteins; QUIKI, quantitative insulin sensitivity check index.

**Table 3:** Multivariate-adjusted Odds Ratios (OR) and 95% Confidence Intervals (CI) of asymptomatic carotid atherosclerosis determined by fish intake and other factors potentially associated with occurrence of atherosclerosis

**Effect**	**OR**	**95% CI**
Age (y)	1.12	1.09 – 1.15
Gender (M vs. F)	1.44	0.92 – 2.24
Smoking status:		
Former vs. never smoker	1.35	0.77 – 2.35
Current vs. never smoker	1.04	0.63 – 1.73
Dietary pattern:		
Intermediate vs. Mediterranean	1.19	0.74 – 1.92
Unhealthy vs. Mediterranean	1.20	0.63 – 2.27
Frequency of fish intake:		
1 vs. <1 servings/week	0.92	0.53 – 1.60
**≥ 2** vs. <1 servings/week	0.52	0.29 – 0.92
Physical activity level:		
Light vs. none	1.0	0.64 – 1.56
Moderate/heavy vs. none	0.71	0.33 – 1.50
Use of statins: yes vs. no	1.27	0.65 – 2.48
Hypertension on treatment: yes vs. no	1.86	1.19 – 2.92
Pulse pressure (mmHg)	1.03	1.01 – 1.04

## Discussion

In this cross-sectional study, we found that high habitual fish consumption was associated with a lower prevalence of asymptomatic carotid atherosclerosis, defined as the presence of carotid plaques and/or increased c-IMT in participants with no clinically known atherosclerotic diseases. Habitual fish consumption was similar in the three dietary patterns considered in this study (Unhealthy, Intermediate, Mediterranean); indeed, the protective association between habitual fish consumption and carotid atherosclerosis was independent of the habitual dietary pattern. Other traditional cardiovascular risk factors, such as age, pulse pressure and hypertension were confirmed as being associated with a higher probability of asymptomatic carotid atherosclerosis. These results are in agreement with data from the Multi-Ethnic Study of Atherosclerosis [26], which showed that the dietary intake of non-fried fish was inversely associated with the prevalence of subclinical atherosclerosis diagnosed on the basis of c-IMT in people without clinical cardiovascular diseases. Similarly, Yamada et al. [27] found that serum long-chain n-3 PUFA concentrations, a biomarker of habitual fish intake, were inversely related to the probability of carotid plaques.

In our study, the condition of high habitual fish consumption corresponded to intakes of at least 2 servings a week. This is in agreement with current nutritional guidelines, which suggest an intake of at least 2 servings a week for prevention of CVD [3,28]. Since carotid atherosclerosis is strongly associated with cardiovascular and all-cause mortality, our findings are also in agreement with studies that indicate that an increase in fish consumption of 1–2 servings a week would reduce CHD mortality (by 36%) and all-cause mortality (by 17%) [7].

Contrary to expectations, we did not find a significant association between carotid atherosclerosis and blood cholesterol concentrations [29]; indeed, cholesterol concentrations were slightly, though not significantly, higher in the group who habitually consumed more fish. This result may suggest that dietary habits are of greater importance than traditional lipidemic cardiovascular risk factors. This is in agreement with the observation of a paradoxical lower cardiovascular mortality despite a high prevalence of cardiovascular risk factors (including cholesterol blood concentrations) in some geographic areas where there is a documented high habitual intake of healthy fats such as olive oil and n-3 PUFA from fish [30,31]. Should this relationship be confirmed, also given the high prevalence of asymptomatic carotid atherosclerosis that we found in our cohort and that is similar to that reported in other studies [32,33], inducing healthy nutritional changes in the population, including fish consumption, may be a plausible strategy for CVD prevention.

Indeed, our results are in agreement with studies demonstrating that elevated consumption of total linolenic acid [34] or eicosapentaenoic acid [35] or docosahexaenoic acid [36] is associated with lower prevalence of carotid plaques or lower carotid intima-media thickness.

We did not find any significant difference or association between fish consumption and any of the measures of glucose homeostasis considered in this study. This result is in agreement with studies that have investigated the association between fish intake and type 2 diabetes, but have provided inconclusive results [37].

The fish consumed by participants in this study was largely (≈ 70%) fresh fish of Mediterranean origin. However, recent ecological investigations have found a high toxicological risk in the marine environment, including the Mediterranean Sea, due to the presence of high concentrations of chemical pollutants, such as heavy metals, that may represent a health risk for humans who consume fish exposed to these contaminants [38-41]. Therefore, despite the favorable health effects detected in our study, caution is advised when the consumption of large amounts of fish (e.g., more than 2 servings per week) is recommended, since no overall assessment of the effects of fish consumption on health is available [42,43].

This study has some intrinsic limitations. First, the sample size is relatively small, which may have blunted the statistical power of the observed associations. Second, given the cross-sectional design of the study, we cannot exclude the possibility of residual confounding. Also, we cannot determine whether differences in the protective effects of fish exist according to differences in fish cooking-procedures (fried or not) or in its provenance (farm-raised fish or their wild counterparts), storage (frozen or fresh) or typology (fatty or lean). Similarly, we have no information on the consumption of fish oil supplements. Though our results suggest that consumption of fish is protective against carotid atherosclerosis, we cannot attribute this potential benefit to specific components of fish. In fact, other substances, apart from n-3 PUFA (which are contained in fish), such as vitamin D or selenium, have been attributed as protective cardiovascular effects. Since we did not enroll a representative cohort of Palermo's population, some bias might be associated with the sampling technique. However, the composition of the cohort we recruited was similar to that reported for the commercial center customers and having also offered the possibility of a thyroid echography check probably induced young people without cardiovascular, metabolic or nutritional known clinical problems to take part in the study. Even the FFQ and the physical activity questionnaire we used in this study were not validated. However, we consider generic half-quantitative data on habitual consumption of different foods as well as habitual physical activity, not amounts of energy expenditure and energy intake or quantitative amounts of each food. This likely reduced inaccuracy.

The strengths of this study are the modality of participant recruitment, which allowed for the characterization of study participants, and the use of a strict ultrasound procedure by two operators, which may have contributed to reducing possible biases.

## Conclusions

Given the vast diffusion of asymptomatic carotid atherosclerosis, our results suggest the possibility of a nutritional strategy to counteract this epidemic condition, though adequate interventional trials will be needed to confirm the role of fish consumption in prevention of cardiovascular diseases.

## Abbreviations

ANCOVA: Analysis of covariance; ANOVA: Analysis of variance; BMI: Body mass index; BP: Blood pressure; c-IMT: Carotid intima-media thickness; CHD: Coronary heart disease; CVD: Cardiovascular disease; FPG: Fasting plasma glucose; GFR: Glomerular filtration rate; HDL: High density lipoproteins; HOMA-IR: Homeostasis model assessment of insulin resistance; LDL: Low density lipoproteins; MDRD: Modification of diet in renal disease study; ORs: Odds ratios; PUFA: Polyunsaturated fatty acids; QUICKI: Quantitative insulin sensitivity check index.

## Competing interests

The authors declare that they have no competing interests.

## Authors’ contributions

The authors’ responsibilities were as follows: S.B. conceived of the study and participated in its design and coordination, carried out ultrasonographic measurements, performed data analysis and interpretation and drafted the manuscript. A.N. performed data analysis and interpretation and contributed to manuscript preparation. D.S. collected the data and contributed to manuscript preparation. S.M. performed data analysis and revised the manuscript. L.C. performed data analysis and revised the manuscript. F.G. interpreted data and revised the manuscript. Se. B. recruited volunteers, managed the clinical study, carried out anthropometric measurements, collected the data, drafted the manuscript. M.L.B critically revised the manuscript for important intellectual content. G.B.R. was responsible for the study, gave data interpretation and revised the manuscript. All authors have read and approved the final manuscript.
